# Sensors for Road Vehicles of the Future

**DOI:** 10.3390/s23010022

**Published:** 2022-12-20

**Authors:** Felipe Jiménez

**Affiliations:** Instituto Universitario de Investigación del Automóvil (INSIA), Universidad Politécnica de Madrid, 28031 Madrid, Spain; felipe.jimenez@upm.es

New vehicles include several systems that improve their safety, comfort, and performance. A key part of these systems is the use of several sensors around the vehicle, capturing information from the vehicle and its surroundings. For this reason, today, the development and implementation of new sensors is crucial to enable using new technologies, improving their measuring capabilities, and providing new information that, up to now, was not necessary but has become essential.

This Special Issue deals with sensors that have been introduced or will be introduced in the near future in road vehicles. Several sensor families are included in this group, such as sensors for assistance systems, sensors for vehicle dynamics, sensors for capturing information from the vehicle’s surroundings, sensors for capturing data from the vehicle interior, sensors for driver supervision, etc. Positioning and digital maps could also be considered as secondary sensors that could provide information, and thus, these challenges will also be taken into account.

Furthermore, new sensors involve new algorithms that need to be implemented for new systems. In this field, we could include perception algorithms (for example, for road or obstacle detection, or for vehicle positioning) and control algorithms for assistance or autonomous applications. In many cases, algorithms involve sensor fusion, and the current trends and solutions in this field are key for obtaining reliable and complete information.

Similarly, although the scope of this Special Issue is not specifically focused on final systems, practical applications supported by these new sensors are also included.

This Special Issue includes fifteen papers. The first group consists of four papers related to sensors for vehicle dynamic measurements and the use of this information for several purposes, including monitoring and the infrastructure of the vehicle.

Firstly, Ref. [1] proposes a wheel odometry model. The parameters of the nonlinear dynamic system are estimated with a Gauss–Newton regression. One of the most relevant problems appears when measurement uncertainty highly corrupts the estimation accuracy, due to the sensors characteristics used in the automotive. The problem is handled with a unique Kalman-filter addition to the iterative loop.

A new method to obtain the wheel speed by using Sin-Cos encoders is presented in [2]. The methodology is based on the use of Savitzky–Golay filters to optimally determine the coefficients of the polynomials that best fit the measured signals and their time derivatives. One of the main advantages of the method is the fact that it implies that there is a low computational load, meaning it can be applied in real-time applications.

The continuous and precise knowledge on road surface properties is relevant information for many systems. Ref. [3] proposes a low-cost approach to solve this issue, based on the analysis of vibrations generated by the tire-rolling movement to classify road surfaces.

A quite different topic is considered in [4]. This paper proposes a local active control method for the reduction in road noise inside a vehicle cabin. A multichannel, simplified. hybrid active noise control system is developed and applied to the rear left seat of a vehicle to assess its attenuation capability, using reference signals provided by accelerometers on the suspensions and bodywork of the vehicle and microphones on the floor of the cabin.

Additionally, the accurate vehicle positioning definition problem and the use of vehicle positioning for assistance systems is the subject of two papers.

Vehicle lateral position (or “where in the lane” problem) is a crucial piece of information for a set of driver assistance systems. A method to estimate the lateral position of the vehicle in its lane using the longitudinal displacement is presented in [5], based on a hyper-frequency interaction between a transceiver module in the vehicle and passive transponders integrated in the road.

Ref. [6] presents a merging assistance system based on vehicle positioning and communications between the two. The system algorithm decides where and when the vehicle can start the merging maneuver in safe conditions and provides the appropriate speed information to the drivers.

Another group of four papers focuses on surrounding perception, improving performance and capabilities of current systems, and providing solutions for onboard systems and cooperative systems.

Obstacle detection is a very fruitful research field, but some enhancements are proposed as some limitations appear in specific scenarios, such as for the detection of some obstacle types or when using some sensors. For example, Ref. [7] proposes a pedestrian detection algorithm with a de-raining module that improves the detection accuracy under various rainy scenarios. Specifically, this algorithm determines the density information of rain and effectively removes rain streaks through the de-raining module.

Additionally, with a focus on pedestrians, but not limited to them, Ref. [8] tries to enhance a Dynamic Obstacle Mapping system by means of improving the perception stage, especially due to the fact that people’s movements are more unpredictable. The extension relies on three key points: LIDAR reflectivity, static and dynamic occlusion detectors, and a tracking stage based on a particle filter.

Camera-based systems can be used for the detection of infrastructure elements or transient conditions, such as adverse weather ones. Ref. [9] presents two vision-based applications for traffic sign recognition and real-time weather alerts, such as for fogbanks. This information is proposed to be used to support operators with road infrastructure maintenance tasks, as well as drivers, giving them valuable information via cooperative services.

In a specific case of the Forward Collision Warning assistance system, an end-to-end deep learning architecture to improve monocular range estimation methods is presented in [10]. In this case, the target range is represented as a weighted sum of a set of potential distances.

The next group of papers deals with autonomous vehicles. The first two papers center on the path controller module, whereas two more papers present a complete architecture of autonomous vehicles, including developments and integration of all control layers.

Ref. [11] suggests two proposals to achieve fast, real-time lane-keeping control for autonomous vehicles. The first proposal is to use a Linear Parameter Varying model to describe the vehicle lateral dynamics, as a trade-off between computational complexity and model accuracy. The second proposal is to use a Dual-Rate Extended Kalman Filter to alleviate the cost of updating the internal state of the filter.

In a similar line, a modular waypoint tracking controller for Robot Operating System (ROS)-based autonomous vehicles is presented in [12]. The controller performs a smooth interpolation of the waypoints and uses optimal control techniques to ensure robust trajectory tracking.

The other two papers present a more global scope than the previous ones. Ref. [13] presents a ROS-based Drive-By-Wire system designed for an autonomous electric vehicle over an open-source chassis. Ref. [14] focuses on the development of a full-stack autonomous vehicle using a limited amount of sensors to avoid creating a very expensive autonomous vehicle with limited application.

Finally, the last paper is focused on applications in which the key point is about V2X communications for proving cooperative services. In this sense, 5G technology is perceived as a disruptive element for enhancing communication capabilities, and the road vehicle environment can take advantage of this. Ref. [15] outlines how highly automated driving in cross-border corridors can be supported. A set of representative use cases and the related communication requirements are identified.

## References

[1] Fazekas M., Gáspár P., Németh B. (2021). Calibration and Improvement of an Odometry Model with Dynamic Wheel and Lateral Dynamics Integration. Sensors.

[2] Alcázar Vargas M., Pérez Fernández J., Velasco García J.M., Cabrera Carrillo J.A., Castillo Aguilar J.J. (2021). A Novel Method for Determining Angular Speed and Acceleration Using Sin-Cos Encoders. Sensors.

[3] Andrades I.S., Castillo Aguilar J.J., García J.M.V., Carrillo J.A.C., Lozano M.S. (2020). Low-Cost Road-Surface Classification System Based on Self-Organizing Maps. Sensors.

[4] Jia Z., Zheng X., Zhou Q., Hao Z., Qiu Y. (2020). A Hybrid Active Noise Control System for the Attenuation of Road Noise Inside a Vehicle Cabin. Sensors.

[5] Mohsen I., Ditchi T., Holé S., Géron E. (2020). Lateral Position Measurement Based on Vehicles’ Longitudinal Displacement. Sensors.

[6] Sánchez–Mateo S., Pérez–Moreno E., Jiménez F. (2020). Driver Monitoring for a Driver-Centered Design and Assessment of a Merging Assistance System Based on V2V Communications. Sensors.

[7] Liu Y., Ma J., Wang Y., Zong C. (2021). A Novel Algorithm for Detecting Pedestrians on Rainy Image. Sensors.

[8] Llamazares Á., Molinos E., Ocaña M., Ivan V. (2020). Improved Dynamic Obstacle Mapping (iDOMap). Sensors.

[9] Iparraguirre O., Amundarain A., Brazalez A., Borro D. (2021). Sensors on the Move: Onboard Camera-Based Real-Time Traffic Alerts Paving the Way for Cooperative Roads. Sensors.

[10] Tang J., Li J. (2020). End-to-End Monocular Range Estimation for Forward Collision Warning. Sensors.

[11] Salt Ducajú J.M., Salt Llobregat J.J., Cuenca Á., Tomizuka M. (2021). Autonomous Ground Vehicle Lane-Keeping LPV Model-Based Control: Dual-Rate State Estimation and Comparison of Different Real-Time Control Strategies. Sensors.

[12] Gutiérrez R., López-Guillén E., Bergasa L.M., Barea R., Pérez Ó., Gómez-Huélamo C., Arango F., del Egido J., López-Fernández J. (2020). A Waypoint Tracking Controller for Autonomous Road Vehicles Using ROS Framework. Sensors.

[13] Arango J.F., Bergasa L.M., Revenga P.A., Barea R., López-Guillén E., Gómez-Huélamo C., Araluce J., Gutiérrez R. (2020). Drive-By-Wire Development Process Based on ROS for an Autonomous Electric Vehicle. Sensors.

[14] Azam S., Munir F., Sheri A.M., Kim J., Jeon M. (2020). System, Design and Experimental Validation of Autonomous Vehicle in an Unconstrained Environment. Sensors.

[15] Velez G., Martín Á., Pastor G., Mutafungwa E. (2020). 5G Beyond 3GPP Release 15 for Connected Automated Mobility in Cross-Border Contexts. Sensors.

